# Asymmetric Distribution of Plasmalogens and Their Roles—A Mini Review

**DOI:** 10.3390/membranes13090764

**Published:** 2023-08-29

**Authors:** Masanori Honsho, Yukio Fujiki

**Affiliations:** 1 Department of Neuroinflammation and Brain Fatigue Science, Graduate School of Medical Sciences, Kyushu University, Fukuoka 812-8581, Japan; 2 Institute of Rheological Functions of Food-Kyushu University Collaboration Program, Kyushu University, Fukuoka 811-2501, Japan; 3 Graduate School of Science, University of Hyogo, Himeji 671-2280, Japan

**Keywords:** plasmalogens, peroxisomes, endoplasmic reticulum, plasma membrane, P4-type ATPase, fatty acyl-CoA reductase 1, homeostasis

## Abstract

Plasmalogens are a unique family of cellular glycerophospholipids that contain a vinyl-ether bond. The synthesis of plasmalogens is initiated in peroxisomes and completed in the endoplasmic reticulum. Plasmalogens are transported to the post-Golgi compartment, including endosomes and plasma membranes, in a manner dependent on ATP, but not vesicular transport. Plasmalogens are preferentially localized in the inner leaflet of the plasma membrane in a manner dependent on P4-type ATPase ATP8B2, that associates with the CDC50 subunit. Plasmalogen biosynthesis is spatiotemporally regulated by a feedback mechanism that senses the amount of plasmalogens in the inner leaflet of the plasma membrane and controls the stability of fatty acyl-CoA reductase 1 (FAR1), the rate-limiting enzyme for plasmalogen biosynthesis. The physiological consequences of such asymmetric localization and homeostasis of plasmalogens are discussed in this review.

## 1. Introduction

Phospholipids are one of the components of biological membranes formed by lipid bilayers, with their hydrophobic fatty-acid chains located in the center of the bilayer and hydrophilic heads facing the aqueous periphery of the bilayer. Phospholipids are not randomly distributed throughout the biological membranes of eukaryotic cell membranes. This asymmetric distribution is most pronounced in the plasma membrane, where phosphatidylethanolamine (PtdEtn), phosphatidylserine (PtdSer), and phosphatidylinositol (PtdIno) are primarily located at the cytoplasmic leaflet of the membrane, whereas phosphatidylcholine (PtdCho), sphingomyelin, and glycolipids are enriched on the external or exoplasmic leaflet [1,2]. The amphiphilic nature of phospholipids hampers the movement of lipids across hydrophobic lipid bilayers. However, the movement of lipids in biological membranes is accelerated by the function of three types of lipid transporters, type-IV P-type ATPases (P4-ATPases), ATP-binding cassette (ABC) transporters, and scramblases [3,4,5]. Of these transporters, the first two translocate specific lipids across the bilayer using the energy of ATP. P4-ATPases act as phospholipid flippases from the exofacial to the cytosolic leaflet, whereas ABC transporters transport lipids in a direction opposite to that of P4-ATPases [6]. The activities of P4-ATPases are required for the formation and maintenance of asymmetric distribution of phospholipids [7,8,9] and are involved in membrane protein regulation [10], phospholipid signaling [11], the maintenance of cell polarity, membrane trafficking [12,13], cytoskeletal dynamics [14,15], and cell differentiation [16].

## 2. Plasmalogens

**Figure 1:**
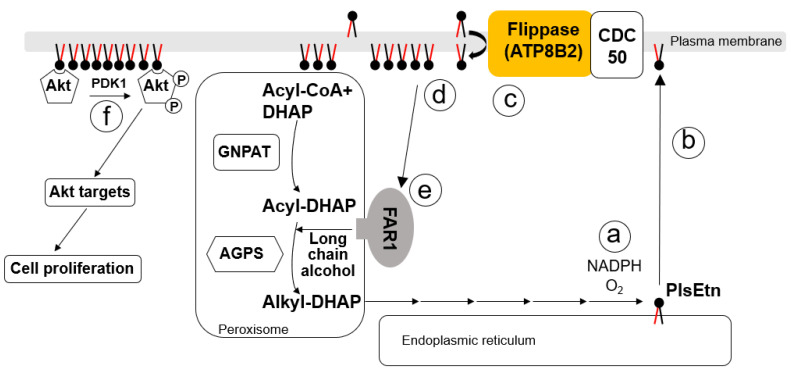
A schematic model of ATP8B2’s role and regulation of plasmalogen biosynthesis. Peroxisomal matrix enzymes, GNPAT and AGPS, sequentially catalyze the synthesis of acyl-DHAP and alkyl-DHAP, respectively, where AGPS catalyzes the formation of alkyl-DHAP by replacing the acyl chain of acyl-DHAP with a long-chain alcohol. FAR1, a peroxisomal C-tail anchored protein, reduces fatty acids to long-chain alcohols. Alkyl-DHAP is reduced to alkyl-glycerol-3-phosphate (Alkyl-G3P), further catalyzed to plasmalogens via the remaining four steps in the ER. The final step for the synthesis of PlsEtn is catalyzed by plasmanylethanolamine desaturase 1 (PEDS1) in a manner dependent on oxygen and cytochrome *b_5_* [18]. (**a**). Plasmalogens are transported to the post-Golgi compartment, including endosomes and plasma membranes, in a manner dependent on ATP, but not vesicular transport [61] (**b**). In the plasma membrane, plasmalogens are preferentially localized in the inner leaflet in a manner dependent on P4-type ATPase ATP8B2, which associates with the CDC50 subunit (**c**). Plasmalogens localized in the inner leaflet of the plasma membrane are sensed (**d**) and the signal monitoring the cellular level of plasmalogens is conveyed to peroxisomes, where the stability of FAR1 is regulated, thereby controlling the synthesis of plasmalogens (**e**). Plasmalogens in the inner leaflet of the plasma membrane contribute to the 3-phosphoinositide-dependent protein kinase 1 (PDK1)-mediated phosphorylation of AKT and participate in cell proliferation through the activation of AKT-downstream proteins (**f**).

Plasmalogens are a type of glycerophospholipid characterized by the presence of a vinyl-ether bond at the *sn*-1 position of the glycerol backbone. Plasmalogens are a major constituent of cellular membranes and are mainly generated by de novo synthesis rather than via dietary intake [17]. The synthesis of plasmalogens is initiated in the peroxisome and completed in the endoplasmic reticulum by the formation of vinyl-ether bonds catalyzed by plasmanylethanolamine desaturase 1 (PEDS1) [17,18,19,20] (Figure 1). Plasmalogen deficiency was found in infants with rhizomelic chondrodysplasia punctata (RCDP) and peroxisome biogenesis disorders (PBDs), where the former is caused by genetic mutation in the gene-encoding proteins required for the synthesis of plasmalogens or factors essential for the transport of the enzyme to the peroxisome. There are five genetic subtypes to underlie RCDP. RCDP1 is the most common type of RCDP and is caused by mutations in the *PEX7* gene encoding the PEX7 receptor that is essential for the import of alkylglycerone phosphate synthase (AGPS) into the peroxisome [21,22,23,24,25]. RCDP2, 3, and 4 are caused by mutations of enzymes, glyceronephosphate-O-acyltransferase (GNPAT), AGPS, or fatty alcohol reductase 1 (FAR1), respectively, which catalyze the synthesis of plasmalogens [26,27,28]. RCDP5 is caused by mutations in the long isoform of PEX5, acting as a cytosolic receptor for PEX7 in the transport of PTS2-AGPS to peroxisomes [29]. These mutations result in a severely reduced capacity of plasmalogen synthesis, hence implying that de novo synthesis of plasmalogens is essential in our health. Moreover, the recent finding that a group of patients with elevated plasmalogen levels in early childhood due to a genetic disorder developed spastic paraplegia clearly demonstrates the physiological importance of plasmalogen homeostasis [30]. Based on these facts, it is now clear that the biosynthesis of plasmalogens significantly contributes to the homeostasis of plasmalogens in the human body. Furthermore, tissue-specific conditional knockout mice showing a defect in plasmalogen synthesis caused by impaired peroxisome biogenesis, including the cerebrum, skeletal muscle, heart, and adipose tissue, show a dramatic decrease in plasmalogens in the respective tissues, highlighting the importance of the local synthesis of plasmalogens in tissues [31,32,33]. In addition to the severe loss of plasmalogens in patients with RCDP or PBDs, moderate reductions in plasmalogens have been reported in neurodegenerative disorders, including Alzheimer’s disease, Parkinson’s disease, and Multiple Sclerosis, as well as cardiovascular diseases such as Barth syndrome and coronary artery disease [34,35,36,37,38,39,40,41,42]. Therefore, an understanding of the molecular mechanisms underlying the regulation of plasmalogen biosynthesis is critical to explore the pathogenesis of diseases associated with reduced plasmalogen levels, as well as to improve disease states and establishing treatments by restoring plasmalogens of the disease.

## 3. Asymmetric Distribution of Ethanolamine Plasmalogen

Plasmalogens are present in all mammalian tissues. Ethanolamine plasmalogens (PlsEtn) are known to be abundant in the brain, heart, neutrophils, and eosinophils, whereas choline plasmalogens (PlsCho) are found in heart and skeletal muscle [17,18,19,20,43]. PlsEtn are thought to be located in the inner leaflet of the plasma membrane in red blood cells and myelin [44,45]. The asymmetric distribution of PlsEtn was further verified by incubating cultured cells with the membrane-impermeable amine reaction reagent 2,4,6-trinitrobenzene sulfonic acid (TNBS), which specifically modifies plasmalogens in the outer membrane leaflet [46]. Approximately 4% of the total plasmalogens were modified by TNBS in HeLa cells, less than the amount of PtdEtn, known to be located in the inner leaflet of plasma membranes [47], implying that PlsEtn are preferentially localized to the cytoplasmic leaflet of the membrane. Given these facts together with a notion of the asymmetric distribution of PtdEtn, P4-ATPase-mediated transport of PlsEtn from the exoplasmic to the cytoplasmic leaflet is expected. This scenario was investigated by lowering the expression of the *CDC50A*-encoding β-subunit of P4-ATPases [46]. The β-subunit forms a heterodimer with several P4-ATPases and plays pivotal roles in the proper folding, organellar targeting, and lipid-flipping of P4-ATPases [48,49,50]. For instance, knockdown of *CDC50A* expression inhibits the incorporation of fluorescent aminophospholipids such as NBD-PtdEtn and NBD-PtdSer [51] due to the dysfunction of P4-ATPases. Similarly, knockdown of *CDC50A* elevates the level of TNBS-modified PlsEtn, suggesting that the asymmetric distribution of PlsEtn in the cytoplasmic leaflet is due to the presence of P4-ATPase(s).

## 4. ATP8B2-Mediated Asymmetric Distribution of PlsEtn

P4-ATPases are exclusively expressed in eukaryotes [3,52]. The human genome encodes fourteen P4-ATPases, and their intracellular localization, substrate specificities, and cellular roles have been explored mainly by using fluorescent glycerophospholipid probes such as NBD-PtdEtn, NBD-PtdSer, and NBD-PtdCho [53,54]. Unfortunately, fluorescent PlsEtn is not commercially available, by which the substrate specificity of P4-ATPase(s) toward PlsEtn has not been investigated. However, potential candidate P4-ATPase(s) responsible for PlsEtn can be picked up thanks to the results of substrate specificity and subcellular localization analysis on the several P4-ATPases identified so far [54,55]. From the basis of expression, intracellular localization, and association with CDC50A in HeLa cells, four P4-ATPases (ATP8B2, ATP10D, ATP11A, and ATP11B) were selected and their flippase activity toward PlsEtn was assessed by monitoring the level of TNBS-modified PlsEtn in cells, where the expression level of respective P4-ATPase was lowered. Knockdown of ATP8B2 expression appeared to enhance the exoplasmic localization of PlsEtn, but not PtdEtn [56], implying that ATP8B2 transports PlsEtn rather than PtdEtn. The substrate specificity of ATP8B2 was assessed using commercially available fluorescent glycerophospholipids, thereby showing that ATP8B2 transports NBD-PtdCho but not NBD-conjugated PtdSer, PtdEtn, sphingomyelin, and glucosylceramide [53]. However, the flippase activity of ATP8B2 toward NBD-PtdCho was lower than that of ATP8B1 and ATP10A, both expressed in the cells used for the analysis of ATP8B2 function, by which impaired PlsCho flippase activity of ATP8B2, if any, is thought to be compensated for by other P4-ATPases such as ATP8B1 and ATP10A [53,57]. Based on these studies, it is concluded that ATP8B2 is most likely the transporter of PlsEtn (Figure 1). Further investigations of mechanistic insight on PlsEtn recognition by ATP8B2 and the identification of other P4-ATPases acting as PlsEtn flippase, if any, are required. As described above, it is unlikely that ATP8B2 transports PtdEtn based on the analyses of flipping activity with NBD-PtdEtn or TNBS modification [56,58,59]. The difference between PlsEtn and PtdEtn, including the presence of a vinyl-ether bond in PlsEtn and/or closer packing of the proximal regions of acyl chains in PlsEtn compared with diacyl counterparts, may be involved in the specific recognition of PlsEtn by ATP8B2 [60]. The development of fluorescent PlsEtn and the structural analysis of ATP8B2 will greatly accelerate the elucidation of the issues related to the topogenesis of PlsEtn.

## 5. Roles of Plasmalogens Located in the Cytoplasmic Leaflet

The involvement of a PlsEtn-sensing step at the cytoplasmic leaflet in the regulation of PlsEtn biosynthesis is proposed from a study in cells that were compromised in the asymmetric distribution of PlsEtn due to a reduced expression of CDC50A [46]. The sensing of PlsEtn is an important step for the feedback regulation of PlsEtn biosynthesis. Two fatty acyl-CoA reductases, FAR1 and FAR2, both localized to peroxisomes and which catalyze a reduction in fatty acids to fatty alcohols, were successfully isolated [62]. Subsequent analyses revealed that FAR1, preferentially catalyzing the synthesis of alcohol containing saturated or unsaturated fatty acids of 16 or 18 carbons [62], is a rate-limiting enzyme of PlsEtn synthesis that is initiated in the peroxisome and completed in the endoplasmic reticulum [20,63,64], whereas FAR2 is involved in the formation of fatty alcohols with carbon chain lengths ≥C24, in meibomian glands, and in the formation of the tear film lipid layer [65]. FAR1 activity is known to be regulated by altering FAR1 protein level in a manner dependent on cellular PlsEtn [20,46,63,64]. In contrast, FAR2 protein level is not changed by the elevation of PlsEtn, suggesting that FAR2 is unlikely to be involved in plasmalogen synthesis [66]. FAR1 protein level is augmented by the dysfunction of *CDC50A* or enzymes responsible for the synthesis of plasmalogens, likely due to the reduction in PlsEtn in the cytoplasmic leaflet [46]. Similarly, an increased FAR1 protein is shown by the knockdown of *ATP8B2*, but not other P4-ATPases such as *ATP10D*, *ATP11A*, and *ATP11C* [56]. These results suggest that the ATP8B2-mediated cytoplasmic leaflet localization of PlsEtn is crucial for the sensing of PlsEtn in the regulation of PlsEtn synthesis.

In addition to the role of cytoplasmically localized PlsEtn in the sensing step of PlsEtn, the asymmetric distribution of PlsEtn is also suggested to be involved in a cellular signaling pathway from the findings of impaired membrane recruitment and subsequent activation of protein kinase B (AKT) in Schwan cells and neurons derived from *Gnpat* knockout (*Gnpat^−/−^*) mice [67,68]. The AKT activation was similarly inhibited in cells by the reduced expression of ATP8B2 [56], reinforcing the role of cytoplasmic-localized PlsEtn in AKT activation (Figure 1). Although any roles of PlsEtn in AKT activation have not been elucidated, PlsEtn is proposed to facilitate the binding of AKT to phosphoinositides through altered membrane properties [69]. AKT activation is thought to be dependent on lipid rafts [70], small platforms composed of sphingolipids and cholesterol in the outer exoplasmic leaflet, which are connected to phospholipids in the inner cytoplasmic leaflet of the lipid bilayer [71]. The enrichment of PlsEtn in lipid rafts is shown in several types of cells [61,72,73]. Moreover, cholesterol, sphingomyelin, and PlsEtn in the enveloped retrovirus HIV, which is derived from the host membranes where virus budding occurs, are enriched as compared with those in host cells [74]. Given these facts together with the asymmetric distribution of PlsEtn, it is more likely that ATP8B2-mediated transport of PlsEtn into the cytoplasmic leaflet plays an important role in the formation of a platform-like domain in the cytoplasmic leaflet that recruits at least AKT. The less-efficient recruitment of AKT in lipid rafts derived from cells with a lower level of plasmalogens was very recently shown in the 4T1 murine mammary carcinoma cell line [75]. The reduced growth rate of cancer cell lines caused by impaired activation of AKT is reported by suppressing ATP8B2-mediated PlsEtn localization in the inner leaflet or reduced levels of dihydroxyacetone phosphate (DHAP), an essential substrate for the synthesis of plasmalogens [56,75]. In addition, impaired AKT activation caused by the absence of plasmalogens in Schwan cells and neurons leads to the abrogation of Schwann cell differentiation and myelination and a lack of control of the position of the axon initial segment, respectively [67,68]. Collectively, these studies indicate that the homeostasis of plasmalogens, involving the sensing of PlsEtn located in the cytoplasmic leaflet of the plasma membrane, plays an important role in the regulation of AKT activity.

In a study of phagocytosis using opsonized zymosan, plasmalogen-deficient cells derived from a macrophage-like cell line RAW264.7 showed a reduced phagocytosis efficiency, as well as reduced activation of extracellular signal-regulated kinase (ERK) p44 and p42, which are returned to normal after incubation of the cells with lyso-plasmenylethanolamine, which is incorporated and reacylated, thus replenishing the cellular plasmalogen pool [76]. The stimulation of macrophages with zymosan enhances arachidonic acid release from glycerophospholipids including PlsEtn, which are enriched in polyunsaturated fatty acids, in a manner dependent on calcium-independent phospholipase A_2_ [77,78], followed by generating eicosanoids, a large family of signaling molecules which play a critical role in inflammatory processes, from arachidonic acid [79,80]. Furthermore, activation of ERK signaling in tumor cells bearing oncogenic Ras seems to be important for ferroptosis [81]. These results, together with findings that plasmalogens containing polyunsaturated fatty acid promote ferroptosis initiated by an inhibitor for the lipid peroxidation repair enzyme glutathione peroxidase 4, expand the possibility of the regulation of signaling pathways by plasmalogen homeostasis [82,83]. We should await further elucidation to understand the role of plasmalogen in ERK activation during phagocytosis and ferroptosis.

## 6. Future Perspective

In recent decades, much has been learned concerning the regulation of PlsEtn synthesis and the roles of PlsEtn in cells and tissues [17,19,20,64,84,85]. The isolation of the gene *TMEM189,* encoding PEDS1, which catalyzes desaturation of plasmanylethanolamine (PlaEtn) to yield PlsEtn, is one of the major recent achievements (Figure 1). According to the data from GEPIA 2 (http://gepia2.cancer-pku.cn/, accessed on: 20 January 2022), the expression of *TMEM189* is upregulated, whereas that of ATP8B2 is reduced in most of the human cancers [56,86]. Moreover, a significantly higher expression of mitochondrial glycerol-3-phosphate dehydrogenase (GPD2) in several cancer tissues than in normal tissues is shown from the transcriptomic comparison study between cancer and normal tissues using the cancer gene atlas (TCGA) TARGET GTEx database [75]. GPD2 catalyzes the synthesis of DHAP from glycerol-3-phosphate, thereby allowing the elevation of DHAP, a substrate of GNPAT. These transcriptional changes in *GPD2*, *TMEM189*, and *ATP8B2* and the resulting metabolic changes may be responsible for the elevated plasmalogens seen in some cancer cells [87,88,89], particularly the decreased expression of ATP8B2, which may reduce plasmalogens in the cytoplasmic leaflet, thereby escaping synthetic inhibition via the plasmalogen-sensing step [56]. The mechanism underlying a transcriptional regulation of *GPD2*, *TMEM189*, and *ATP8B2* in cancers remains to be explored.

The generation of *Peds1*-deficient mice and subsequent analysis of ether lipid homeostasis show unaltered FAR1 protein levels in the cortex despite the absence of plasmalogens [90], suggesting that PlaEtn is transported to the inner leaflet and its levels are sensed to regulate ether lipid biosynthesis, as in the case of PlsEtn. Based on these results, it is expected that asymmetrically localized PlaEtn by means of ATP8B2 is sensed, as in the case of PlsEtn, suggesting that ATP8B2 transports at least three phospholipids, including PlsEtn, PtdCho, and PlaEtn, where the ability of transport of the first two phospholipids was shown by either the TNBS modification method or NBD-PtdCho [31,34]. A study addressing the substrate specificity and structural analysis of ATP8B1, which has an 83% similarity to ATP8B2, revealed that several substrates, such as PtdCho, PtdSer, PtdIns, and plasmanylcholine, are transported by ATP8B1 [91]. Therefore, careful analyses involving structural analysis of ATP8B2 are indeed necessary to fully understand how three phospholipids are transported by ATP8B2.

Recent studies addressing the potential function of plasmalogens in the activation of the cellular signaling pathway showed an activation of ERK- and AMP-activated protein kinase (AMPK) by exogenously added plasmalogens [92,93]. The physiological consequence of plasmalogen-mediated activation of the signaling pathway is not uncovered, although a beneficial effect on cognitive function is reported upon oral administration of plasmalogens [94,95,96]. Importantly, plasmalogens are secreted from keratinocytes, digested by secretory phospholipase A group IIF sPLA-IIF, and the resultant lyso-plasmalogens exacerbate psoriasis [97]. The way that plasmalogens are secreted from keratinocytes remains unknown, whereas secretion associated with vesicles such as exosomes is favored, as is the enrichment of plasmalogens in exosomes [98,99]. Thus, plasmalogens may be translocated from the cytoplasmic leaflets of the plasma membrane to the outer leaflets of vesicles, whereby the plasmalogens are digested by sPLA-IIF. Therefore, it is important to investigate whether ATP8B2-mediated topogenesis of plasmalogens is canceled prior to the formation of multivesicular bodies and/or exosomes in keratinocytes in order to further understand the physiological role of ATP8B2-mediated asymmetric distribution of plasmalogens.

**Figure 2:**
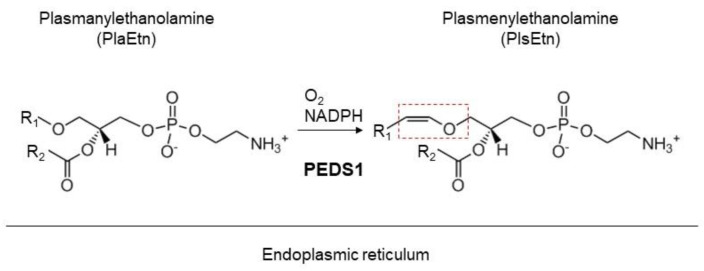
The final step of the plasmalogen synthetic pathway. In the last step of plasmalogen synthesis, plasmanylethanolamine desaturase 1 (PEDS1) catalyzes the formation of vinyl-ether bond (dashed square) of plasmenylethanolamine (PlsEtn) from plasmanylethanolamine (PlaEtn) using NADPH and oxygen as cofactors.

The physiological significance of the findings obtained from the assessment of FAR1 stability in *Peds1*-knockout mouse has not yet been explored [90]. It is plausible that PlaEtn, rather than PlsEtn, is preferentially synthesized under some conditions, such as a hypoxic state, due to the limited available oxygen which is required for the activity of PEDS1 (Figure 2) [100,101]. Moreover, several genes encoding enzymes for the synthesis of PlsEtn and proteins called peroxins required for peroxisome biogenesis are shown to be essential for cell survival under hypoxic conditions from genetic screening in several types of cells [102]. In contrast, PlsEtn is not essential for the survival of cells in normoxia because several plasmalogen-deficient Chinese hamster ovary mutant cells and patient-derived fibroblasts, including peroxisome biosynthesis-deficient mutant cells, have been isolated in normoxic conditions [26,27,103,104,105,106]. Therefore, PlaEtn rather than PlsEtn may play a major role in cell survival in hypoxic compartments where stem cells are localized. It is clear that we should await future studies addressing the homeostasis and functional roles of ether lipids, including PlsEtn and PlaEtn, to fully understand the physiological roles of ether lipids.

Finally, the way for the replacement or supplementation of plasmalogens in the brain has not been established yet [17]. Plasmalogen replacement in peripheral tissues has been shown to be possible, at least in animals, via oral administration of alkylglycerol and synthetic plasmalogens containing proprietary cyclic phosphoethanolamine groups [107,108]. In addition, the synthetic plasmalogen normalizes the hyperactive phenotype associated with a reduction in neurotransmitters, particularly monoamine, in the brain of heterozygous *Pex7*^hypo/null^, resembling milder RCDP1 [108]. Similarly, 1-O-tetradecyl glycerol (1-O-TDG) treatment rescued myelination in plasmalogen-deficient oligodendrocytes in mutant mice [109]. However, the restoration of plasmalogens in the brain upon administration of the synthetic plasmalogen was not observed and the level of plasmalogens in the brain was not described by the treatment of 1-O-TDG despite the elevation of plasmalogens in mouse fibroblasts cultured with 1-O-TDG [108,109].

Thus, these molecules, such as alkylglycerol, synthetic plasmalogens, and 1-O-TDG, which have the ability to rescue dysfunction and developmental defects in the brains of mice with impaired plasmalogen homeostasis, are potential candidates for further improvement to normalize plasmalogen levels in the brain. Moreover, small molecule(s) that enhance the expression of *GPD2* and *FAR1* or that are inhibitors for ATP8B2 may also be useful for elevating plasmalogen levels in the brain.
